# Research on the improvement method of imbalance of ground penetrating radar image data

**DOI:** 10.1038/s41598-025-87123-3

**Published:** 2025-01-22

**Authors:** Ligang Cao, Lei Liu, Congde Lu, Ruimin Chen

**Affiliations:** Key Laboratory of Earth Exploration and Information Techniques of Ministry of Education, Chengdu University of Technology, Chengdu, 610059 Sichuan China

**Keywords:** Ground Penetrating Radar (GPR), Generative Adversarial Network (GAN), Unbalanced dataset, Geophysics, Civil engineering

## Abstract

Ground Penetrating Radar (GPR) has been widely used to detect highway pavement structures. In recent years, deep learning techniques have achieved significant success in image recognition, which is potentially relevant for interpreting ground-penetrating radar data. This is because the various types of damage develop at different levels and in different quantities. So the number of datasets of various types of road injuries is not balanced. This leads to poor accuracy of deep learning for injury classification. And the cost of collecting a large amount of data in the field is higher. The aim of this paper is to improve classification accuracy at a lower cost relative to field collection, we propose a damage data expansion method based on generative adversarial network, which consists of encoder and a generative adversarial network. We have made a number of improvements to the generator and discriminator, as well as to the newly added encoder. All of these improvements have improved the generation results in terms of metrics. So that the network can stably generate damage samples with a small number of samples to improve the classification network’s accuracy. The effect on accuracy by varying the proportions of different kinds of samples and traditional expansion methods is also explored. The improvement of the classification network accuracy and FlD metrics illustrates the better performance of the proposed method.

## Introduction

Road traffic is one of the most basic modes of transportation in modern society, and good road conditions are the basis for ensuring safe and smooth road traffic. GPR technology has gradually become a popular method of pavement damage detection due to its advantages of being fast, efficient and non-destructive. It has become a key issue to accurately find out the damage from a large amount of data. With the development of deep learning, the use of deep learning in ground-penetrating radar data interpretation has gradually begun to reduce labor costs. Zhen Liu et al. proposed a method to identify internal defects in asphalt pavements by combining YOLO sequences with 3D ground-penetrating radar images, and compared the effectiveness of conventional and ground-penetrating radar inspections by evaluating the repair benefits. GPR detection was implemented to obtain images of hidden defects. This is because efficient and accelerated models with acceptable accuracy are needed for real-time pavement crack detection tasks, but are difficult to achieve. In a work, Tianjie Zhang et al. proposed a customized deep learning model architecture called Efficient Crack Segmentation Neural Network (ECSNet) for accelerating real-time pavement crack detection and segmentation without compromising performance. It maintains a good balance between accuracy and efficiency metrics^1,2^. The intersection of ground-penetrating radar and deep learning has now been explored well by many researchers. This includes, but is not limited to, automatic detection, denoising, and expanding data. Many novel networks and modules are used. This manuscript hopes to expand the ground-penetrating radar data using the changed GAN network to get better and finer ground-penetrating radar data closer to the real world. It is used to solve the imbalance problem of ground-penetrating radar data, and ultimately improve the classification accuracy.

In supervised deep learning, the training of models often requires a large amount of labeled data, and even the accuracy of the final model is more closely related to the amount of data used in the training of the model, in other words, the amount of data has a large impact on the results of the model^3^. In addition, most of the training datasets used for ground-penetrating radar target detection networks consist of only a few thousand data, and most of the data were collected in the same experimental environment, resulting in a high degree of similarity between the data and the limited amount of valid data^4^. Deep learning is about learning the distribution of data in a high-dimensional space, so it is desirable that the training data cover all distributions of this type of data as much as possible. So that the model will misjudge less when it encounters different types of data later. Generating data is a research direction that needs to be addressed. Commonly used methods for ground-penetrating radar data enhancement include traditional methods, simulation software-based methods, and deep learning model-driven methods^5^.

The first conventional data enhancement method involves stretching, compressing, translating, flipping horizontally and vertically, and inverting horizontally the ground-penetrating radar B-scan data. What is referred to as “data enhancement” in traditional methods is quite different from “data generation”. Data enhancement involves only image processing of an existing dataset and does not generate new target data^6^. The second method is a type of software such as GprMax, GprMax software is a forward modelling tool^7^. However, generating GPR data that is highly similar to the desired and actually collected data requires extremely fine modeling. It is also computationally intensive, time-consuming, difficult to generate, and requires high computer equipment to generate large-scale data^4,8^. The last category is deep learning model-driven approaches that utilize GAN networks to generate data. This has been explored a lot and is advantageous in many ways. For example, the computational speed is fast, the generated data is closer to reality, and there is no need to change the model frequently in order to generate different data.

There is a more serious imbalance problem in ground-penetrating radar data, which seriously affects the accuracy of the classification network. There exists a large amount of unbalanced data in other domains, such as cloudy and sunny conditions in the weather, the number of bad bank loans^9^, and the number of people suffering from cancer in the medical field^10^. Categorizing this data is often encountered, where we call a large amount of data the majority class and the class with a small amount of data the minority class^11^. Although the minority classes are small in number, they may be more critical and are the targets we would prefer to find out, and misclassification is fatal. Currently, minority class detection and learning based on unbalanced data is not just being focused on as a challenge in the field of data mining but has become a cross-research challenge^12^. When learning in such highly unbalanced data, classifiers can easily favor the majority class^13^. Meanwhile, the purpose of feature selection is to reduce redundant features, and retaining features with strong discriminative power can improve the performance of the classifier as well as prevent the classifier from overfitting whereas, in the case of unbalanced data, the features selected by traditional feature selection methods are more skewed towards the majority class while ignoring the minority class. For example, in cancer detection, omissions in a few categories will lead to extremely serious consequences. Therefore, to train a classification model that is more suitable for the unbalanced data, it is particularly important to process the unbalanced data. In response to the above problems, a series of studies on cases related to unbalanced data have appeared, such as studies based on resampling unbalanced data^14^, algorithms based on unbalanced datasets^15^, and some multi-categorization studies^10,16,17^. This issue was also addressed by Tianjie Zhang et al. in their work, where they used an integrated APC-GAN and Attu-Net framework as a solution for pixel-level automatic segmentation of pavement surface cracks for small training datasets^18^.

Deep learning has been a hot topic in the direction of computer research in recent years. The most recent major event that pushed deep learning forward was the proposal of the convolutional neural network, which simply puts in labeled target data and extracts features to learn on its own^19^. Generative models in machine learning have historically developed less dynamically than classification detection models, but this has changed with the advent of Generative Adversarial Networks (GANs)^20^. GANs can generate virtual images from training data that do not exist in reality. By adding labels to the inputs of generators and discriminators or introducing a set of generators and discriminators, improved GANs can learn nonlinear mapping relationships between different domains, enabling end-to-end image transformations^21^. The GAN framework can be used to perform several other ground-penetrating radar data processing, such as removing clutter from ground-penetrating radar images and performing ground-penetrating radar simulations to enhance the data^22–26^.

## Other methods

Data imbalance can cause the model to have a preference for the majority of categories and ignore the features of a few categories, thus affecting the generalization ability and classification performance of the model. There are currently some other ways to address data imbalance by increasing samples^27–29^.

Resampling techniques: balancing the number of samples from different categories in a dataset by resampling minority category samples or undersampling majority category samples. Oversampling can increase the number of samples in the minority category but can also lead to overfitting. Undersampling can reduce the number of samples in the majority category but may also lead to loss of information.

Synthesizing samples: for image data, new samples can be synthesized using data enhancement techniques that increase the number of samples in a few categories. Data enhancement techniques include methods such as rotating, cropping, scaling, flipping, changing colors, adding noise, etc., which can increase the diversity and robustness of the data. However, ground-penetrating radar data contains direction and position information and is a single-channel grayscale image, so rotation, scaling, and color transformation cannot be applied.

### Method

In this paper, we propose a GAN-based framework for generating ground-penetrating radar damage data in an attempt to solve this problem. The basic structure of the GAN used in this paper is elaborated below.

## Network architecture

**Fig. 1:**
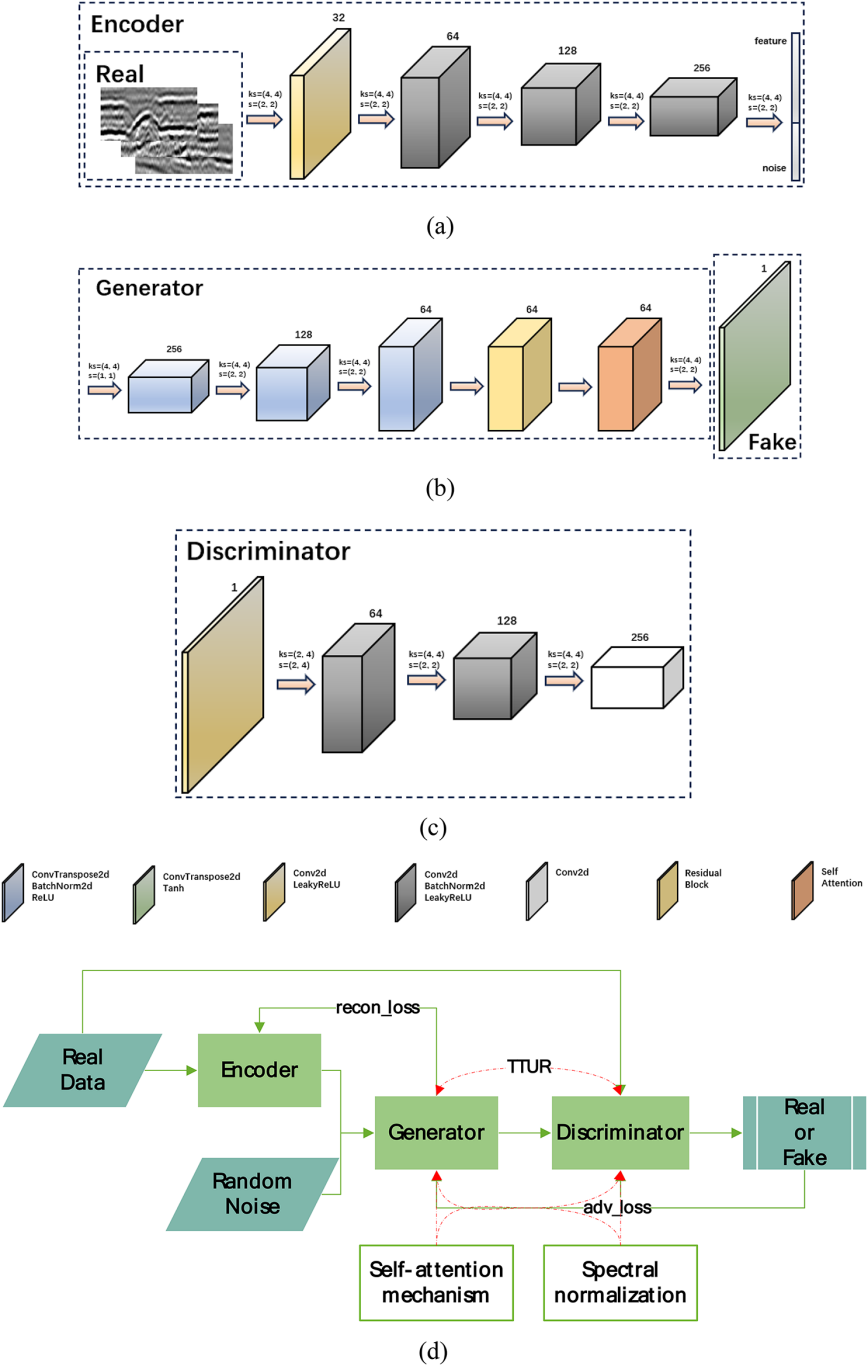
Figure 1. Network structure diagram (a) Encoder structure (b) Generator structure (c) Discriminator structure (d) Overall structure (different colors represent parts of different structures, each part of the structure is illustrated in the Figure)

The network architecture used in this paper consists of a generator network G, a discriminator network D, and an encoder network E. The G network generates the target image while the D network discriminates the generated object to correct the effect of the generator^30^. The E network encodes the real data and then feeds it into the G network. The results of the G network are computed with the real data to get the loss to help improve the G network. Because the actual image size of the ground-penetrating radar highway damage averages around 64, we changed the convolution kernel size and adjusted the number of up-samples to generate images with our specified resolution. Damage image size resolution is low, we scaled down the number of layers in the generator and discriminator, and adding extra layers does not give better results. The structure is shown in Fig. 1.

The encoder is divided into four parts, the original real data is extracted layer by layer and turned into feature, and random noise together into the generator for training. The generator is divided into five parts, after training to get the fake image. The discriminator is divided into three parts, after getting the real image or fake image, the result is obtained through the discriminator network, and then the result is returned to the generator and discriminator to correct the training. The network structure is shown in Fig. 1.

## Two-timescale update rule

The Two-Timescale Update Rule (TTUR) is a method for training GANs that sets different learning rates for the discriminator and generator separately^31–33^. This allows the discriminator to converge to a local optimum faster, while the generator adapts more slowly to changes in the discriminator. TTUR improves the training stability and generation quality of GANs and avoids the problems of gradient vanishing or pattern collapse. This technique is designed to allow the generator and discriminator to choose different learning rates. In the paper where TTUR was first introduced^31^, the authors provided mathematical proof that the algorithm converges to a Nash equilibrium and demonstrated that some of the better-known GANs (DCGAN, WGAN-GP^34^) were implemented using different learning rates with state-of-the-art results. When using TTUR, it may be noticed that the generator has a larger amount of loss.

The basic idea of TTUR is to use stochastic likelihood theory to describe the training process of a GAN in terms of two stochastic differential equations coupled with each other, and then to show that under certain assumptions, TTUR can converge the GAN to a stable local Nash equilibrium. The implementation of TTUR can be done by using different optimization algorithms, such as stochastic gradient descent (SGD), Adam, etc. TTUR can also be used in conjunction with other techniques to improve the GAN such as Wasserstein distance, gradient penalty, spectral normalization, etc.

## Spectral normalization

Spectral normalization is a technique for generative adversarial networks that constrains the number of spectral paradigms of the weight matrix of each layer of the discriminator’s network, thus ensuring that the discriminator satisfies Lipschitz continuity. This enhances the stability of the generative adversarial network during training and avoids the problem of vanishing gradients or collapsing patterns. The spectral parameter is the maximum singular value of the matrix, which reflects the maximum stretch factor of the matrix concerning the input. Spectral normalization is achieved by dividing the weight matrix by its spectral vanity after each update so that its maximum singular value is 1. In this way, the maximum stretch factor of each layer of the network on the input does not exceed 1, thus satisfying the requirement of Lipschitz continuity. The implementation of spectral normalization can be done by using the power iteration method to approximate the spectral norm of the solving matrix, which can reduce the consumption of computational resources^35^.

In some papers, such as that of Self-Attention GAN^36^, it is shown that spectral normalization is a special kind of normalization applied to convolutional kernels, which can greatly improve the stability of training. It was initially used only in discriminators and was later shown to be effective if used in the convolutional layer of a generator as well. In this paper, spectral normalization has been added to both the generator and discriminator.

## Self-attention mechanism

Many neural networks have added attention mechanisms to improve their performance. The difference between the attention mechanism and the self-attention mechanism is that the traditional attention mechanism occurs between the elements of the target and all the elements in the source. This simply means that the computation of weights in the attention mechanism requires the participation of the target. That is, in the Encoder-Decoder model, the computation of attention weights requires not only the implicit state in the encoder but also the implicit state in the decoder.

The self-attention mechanism is not an attention mechanism between the input statement and the output statement, but an attention mechanism that occurs between elements within the input statement or between elements within the output statement. For example, when calculating the weight parameters in the transformer, converting the text vector into the corresponding KQV only requires the corresponding matrix operation at the source, which does not use the information in the Target^37,38^.

The purpose of introducing the self-attention mechanism is that the input received by the neural network is a lot of vectors of different sizes, and there is a certain relationship between the different vectors, but the actual training cannot give full play to the relationship between these inputs and lead to very poor model training results^39^. For example, the machine translation problem (sequence-to-sequence problem, the machine decides how many labels), the lexical labeling problem (one vector corresponds to one label), the semantic analysis problem (multiple vectors correspond to one label), and other word processing problems. This problem of the inability of fully connected neural networks to establish correlation for multiple related inputs is solved by the self-attention mechanism, which tries to make the machine notice the correlation between different parts of the whole input.

### Loss function

The loss function is composed of two parts: generator loss and discriminator loss. The generator loss is the difference between the output of the generator network and the real image and is usually computed using Mean Square Error (MSE) or Binary Cross Entropy. The discriminator loss is the difference between the output of the discriminator network and the labels of the real and generated images and is usually calculated using Binary Cross Entropy as well.

1 $$\:\begin{array}{cc}{\mathrm{L}}_{D}&\:=-{\mathrm{E}}_{x\sim\:{p}_{\text{data}}\left(x\right)}\left[{\rm log}D\right(x\left)\right]-{\mathrm{E}}_{z\sim\:{p}_{z}\left(z\right)}[{\rm log}(1-D(G\left(z\right)\left)\right)]\\\:{\mathrm{L}}_{G}&\:=-{\mathrm{E}}_{z\sim\:{p}_{z}\left(z\right)}\left[{\rm log}D\right(G\left(z\right)\left)\right]\end{array}$$

Where $\:D\left(x\right)$ denotes the output probability of the discriminator on the real

Image x,$\:D\left(G\right(z\left)\right)$ denotes the output probability of the discriminator on the generated image $\:G\left(z\right)$, $\:{p}_{\text{data}}\left(x\right)$ denotes the distribution of the real image, and $\:{p}_{z}\left(z\right)$ denotes the distribution of the random noise^40^.

Because of the small amount of data and the number of training times, it is easy to fall into overfitting.L2 regularization is a method of preventing overfitting, which can keep the weights of the model with small values, thus making the model smoother and reducing the fitting to the noisy data.L2 regularization is based on the principle of adding a regularization term in the loss function to penalize the magnitude of the weights. The effect of L2 regularization can be divided into three aspects: (1) L2 regularization can limit the complexity of the model and prevent the model from being too flexible, thus improving the generalization ability of the model; (2) L2 regularization can make the distribution of the weights more uniform, avoiding the situation where some weights are too large or too small, thus improving the stability of the model; (3) L2 regularization can make the values of the weights smaller, so that the activation function can work in the linear region, thus avoiding the problem of vanishing or exploding gradients and improving the training efficiency of the model^41,42^. The changed loss function can be expressed as:

2 $$\:{\mathrm{L}}_{\text{reg}}={\mathrm{L}}_{D}+{\mathrm{L}}_{G}+\frac{\lambda\:}{2}{\sum\:}_{i}{w}_{i}^{2}.\:$$

Where $\:{w}_{i}$ denotes the ith weight of the model and $\:{\sum\:}_{i}{w}_{i}^{2}$ denotes the sum of squares of all weights.

## Experiment

### Classification network

**Fig. 2:**
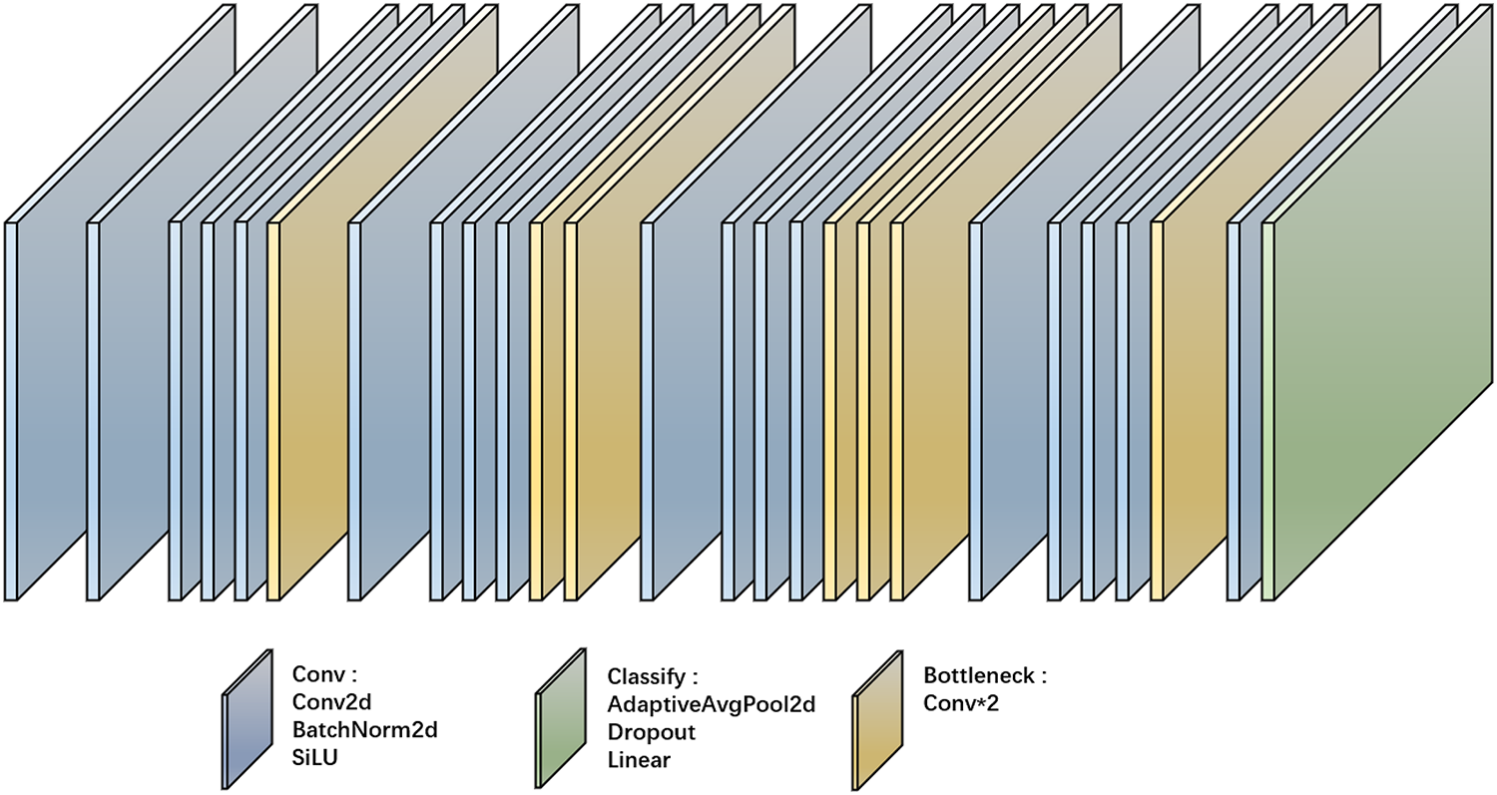
Yolov5s-cls network structure

The classification network used is Yolov5s-cls, the network structure is as follows in Fig. 2, and the main purpose is to verify whether the generated data has improved the classification accuracy.

The network structure is shown in Fig. 2, and the specific structure of each layer is located below Fig. 2.

YOLOv5 is the world’s most popular vision AI, representing Ultralights’ open-source research into the future of vision AI methods, combined with lessons learned and best practices amassed over thousands of hours of research and development. yolov5 is designed to be fast, accurate, and easy to use, making it an excellent choice for a wide range of object detection, image segmentation, and image classification tasks^43^.

### Dataset

**Fig. 3:**
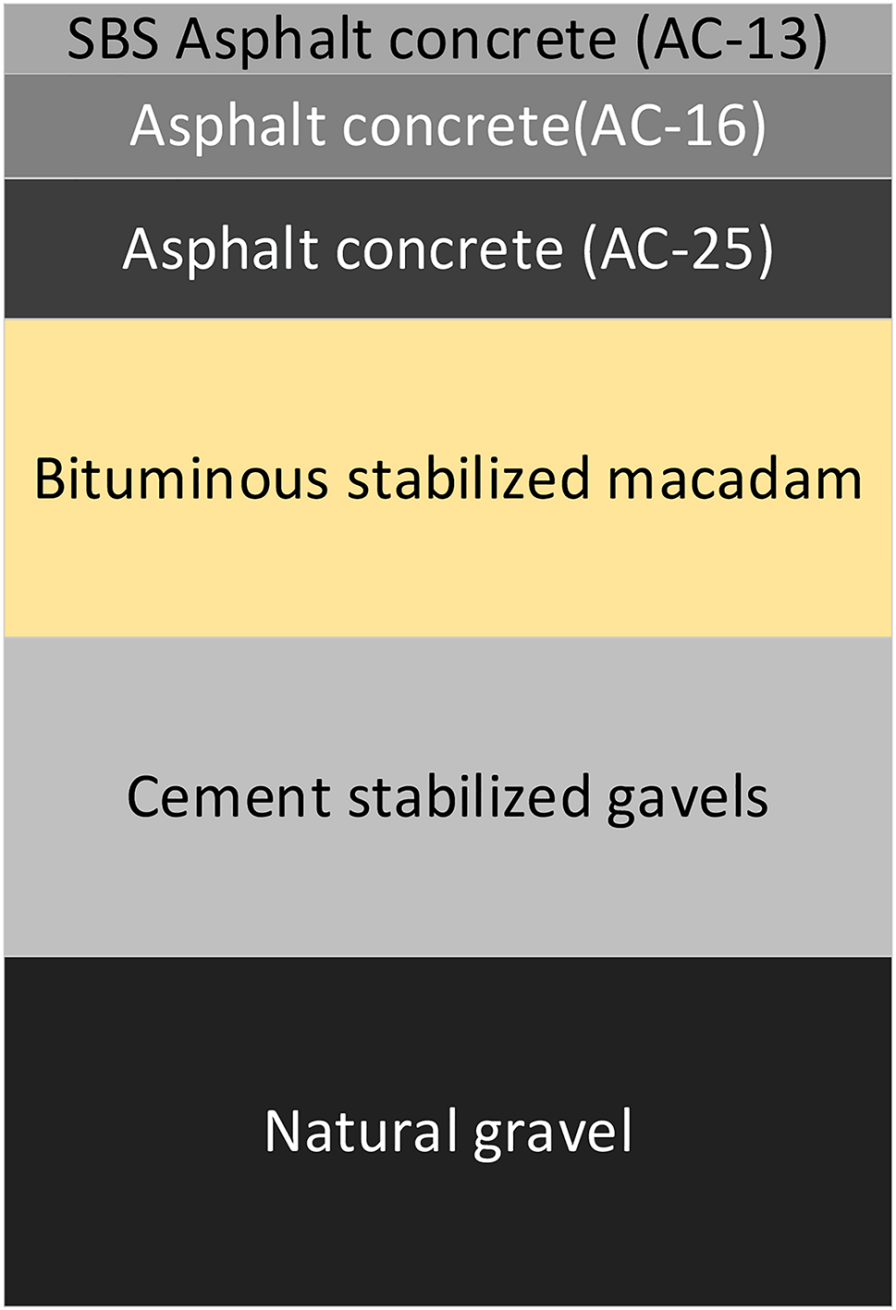
Roadbed structure

The data used were measured ground-penetrating radar images of a domestic highway, measured image acquisition for the vehicle MALA GX750 model of ground-penetrating radar instrument acquisition, acquisition of the car’s speed is 55 km/h, the center frequency is 750 MHz, the coupling mode for the air-coupled, the road structure of the uppermost asphalt surface layer (4 cm + 6 cm + 8 cm), the middle of the water-stabilized grass-roots level (18 cm + 18 cm), the lowest is the sub-base level (20 cm), usually we don’t go to the differentiation of asphalt surface layer of the three layers of the structure because of the bonding situation is very good and will not be problematic, the road structure is as follows Figure. 3.

The raw data were processed in 8 steps, and the detailed data processing included: (1) road interpolation, averaging the length of the detection data into the length of the actual pile number on the site, which is convenient for the location correspondence; (2) subtracting average (de-distortion), the filtering parameter was set to 1.33; (3) static correction, deleting more than the air signals above the road surface; (4) energy decay, suitable for determining the pavement structure of internal damages, adjusting the scaling value, parameters to the maximum amplitude of the longitudinal waveform in the window; (5) subtracting average, the horizontal signal removal ability is strong; (6) anti-folding product, generally high attenuation of the signal will be a continuous “black and white signals,” optional The band-pass filter (frequency cutoff) and the burr noise removal were used.

Next, manual labeling was performed, and the lesions were divided into four categories: loose layers, loose structure, porous, and poor interlayer. Among them, there were 8243, 400, and 2708 damage images for interlayer relaxation, porous, and interlayer failure, respectively, but only 138 images for loose structure. As shown in Table 1.

**Table 1:**
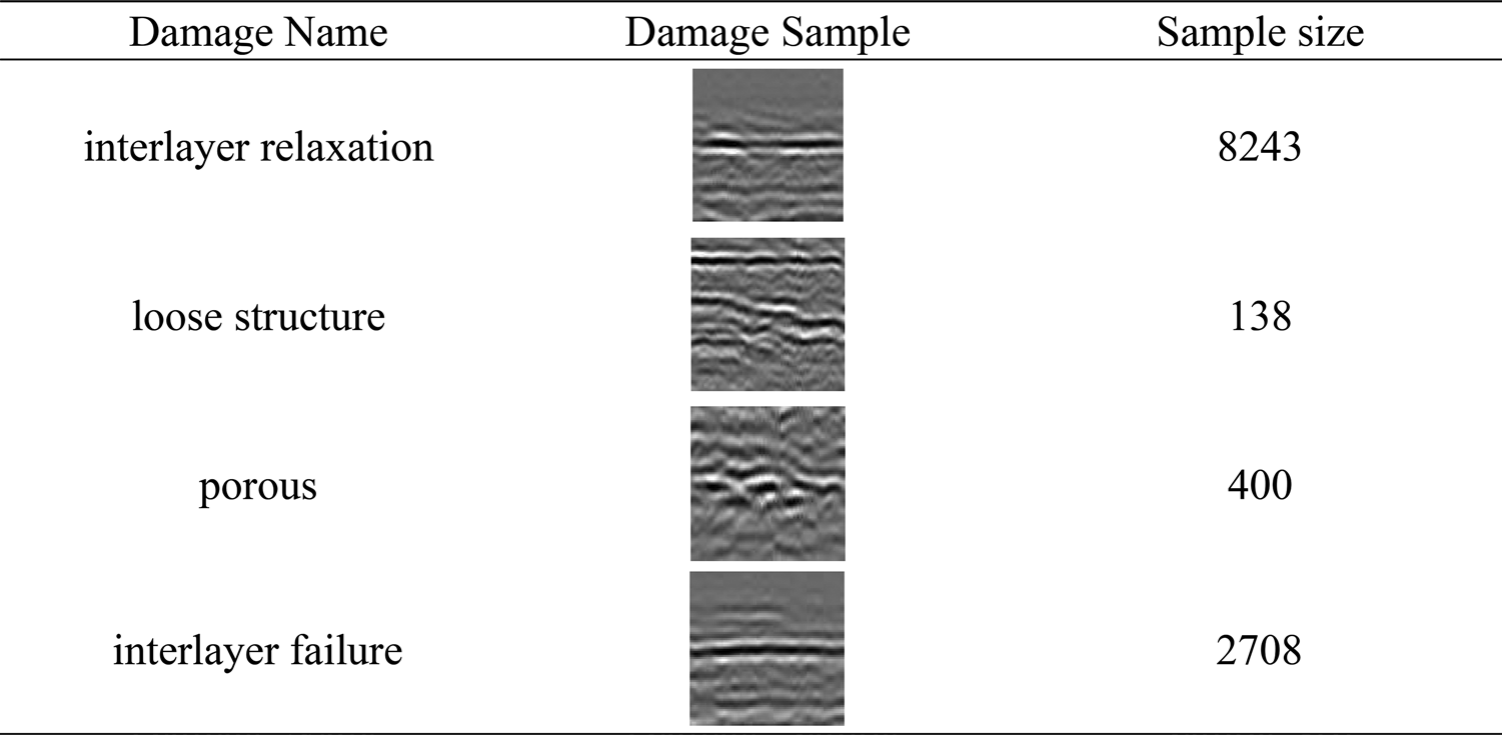
Damage samples and numbers

We trained 20,000 epochs on the proposed network in this paper and other used network. We initiated the training process using an Adam optimizer with a learning rate of 0.0001 and a random noise of 128*1*1. The experiments were performed on a desktop computer with a CPU of Intel i7-13700 K, a graphics card of NVIDIA RTX 4090, and a memory of DDR4 128GB 3200 MHz.

**Fig. 4:**
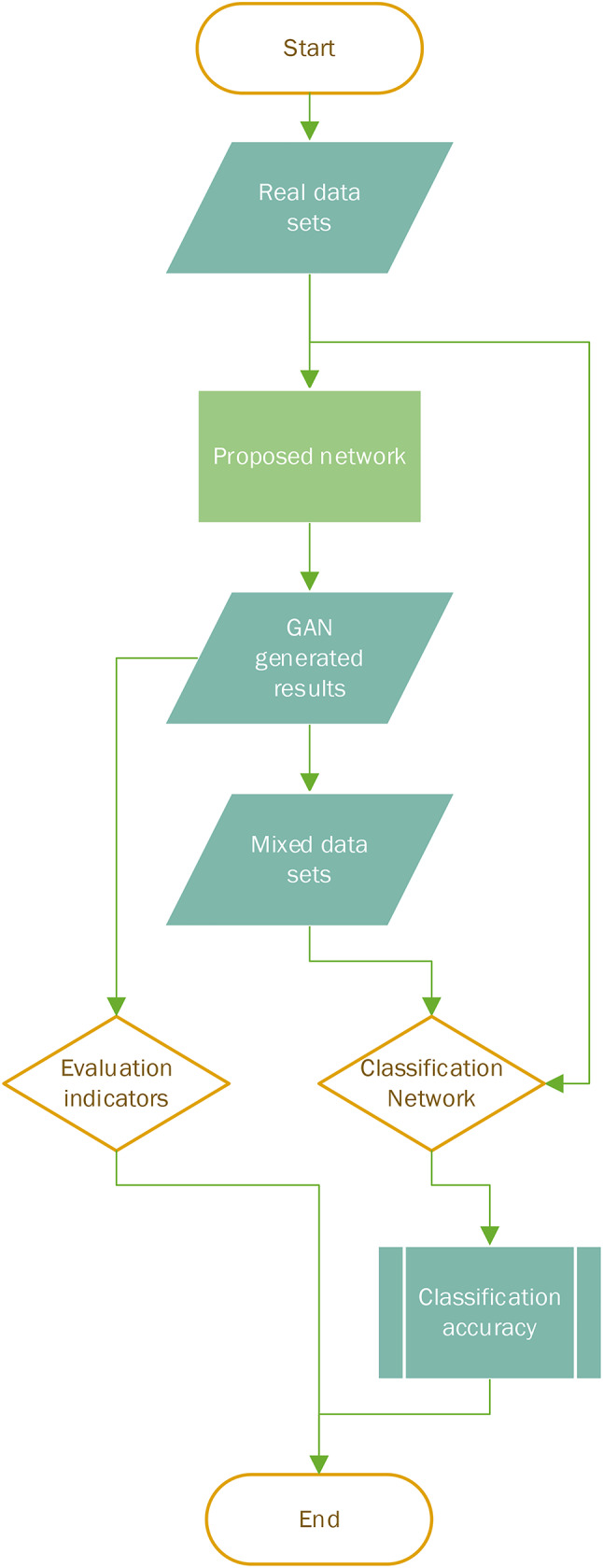
Experimental flow chart

The GAN network uses the original dataset as the material to generate the results, and the obtained data and the original dataset form a synthetic dataset, comparing the classification accuracy of the classification network on each dataset to obtain the evaluation of various datasets and then assess the effectiveness of various methods. The flow chart of the experiment is as follows Figures. 4.

**Fig. 5:**
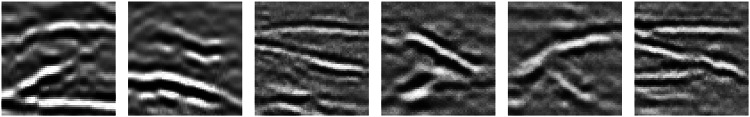
Original and generated results (the first left and second left are the original Figures; the rest are the generated results)

Figure.5 shows some of the generated results.

The generated results are visually good. It is similar in form and texture to the original image. Original and generated results is shown in Figures. 5.

### Evaluation indicators

T-SNE (T-distributed stochastic neighbor embedding) is a dimensionality reduction technique for visualizing high-dimensional data, which maps each data point into a two- or three-dimensional space so that similar data points are close together and dissimilar data points are far away^44,45^. T-SNE is based on a modification of the SNE algorithm, which uses a t-distribution to model the similarity between data points in a low-dimensional space. T-SNE’s algorithm consists of two main steps. In the first step, T-SNE constructs a probability distribution that represents the similarity between data points in a high-dimensional space, where similar data points are assigned a higher probability and dissimilar data points are assigned a lower probability. In the second step, T-SNE defines a similar probability distribution that represents the similarity between data points in the low-dimensional space and minimizes the KL scatter between the two distributions, thus determining the location of the data points in the low-dimensional space. T-SNE can be applied to data visualization in a variety of domains, e.g., genomics^46^, natural language processing^47^, music analysis^48^, and cancer research^49^.

Typically, Initiation Score (IS) and Fréchet Initiation Distance (FID) are two widely accepted metrics for evaluating the performance of GAN models. The IS metric directly evaluates the generated image itself by computing its entropy^50^. On the contrary, the FID measure computes the similarity between the generated image and the field image and therefore outperforms the measure. the FID (Fréchet Inception Distance) is a metric that evaluates the quality and diversity of the generated images. It uses a pre-trained Inception v3 model to extract the feature vectors of the generated image and the real image, and then calculates the mean and covariance matrices of the two sets of vectors, and then measures the similarity between the two multivariate normal distributions using the Fréchet Distance. The smaller the FID, the closer the generated image is to the real image^51,52^.

FID calculation process: import the pre-trained Inception model and extract the features of the real image and the generated image. Calculate the Fréchet distance between the features of the real image and those of the generated image, the smaller the Fréchet distance, the more similar the distribution between the generated image and the real image. FID is widely used to evaluate the performance of generative models. It quantifies the Fréchet distance between the quality of the generated image and the real image and helps researchers improve the training process of the generative model. FID can also be used to compare the performance between different generative models and to evaluate the progress of the generated image in different training stages^53,54^. FID is given by the following equation. 

3 $$d_{FID} \left( x,g \right) = \parallel \mu_{x}-{\mu}_{g}\parallel^{2} + Tr \left[ {\sum}_{x}+{\sum}_{g}-2{\left({\sum}_{x}{\sum}_{g} \right)}^{\frac{1}{2}} \right].$$

Where $\:{\sum\:}_{x}$ and $\:{\sum\:}_{g}$ are the covariance matrices of the field and the generated image, Tr is the trace computed from the sum of the elements along the main diagonal of the square matrix, $\:{\mu\:}_{x}$ and $\:{\mu\:}_{g}$ are the dimensional activations of the field image and the generated image, respectively.

### T-SNE results

**Fig. 6:**
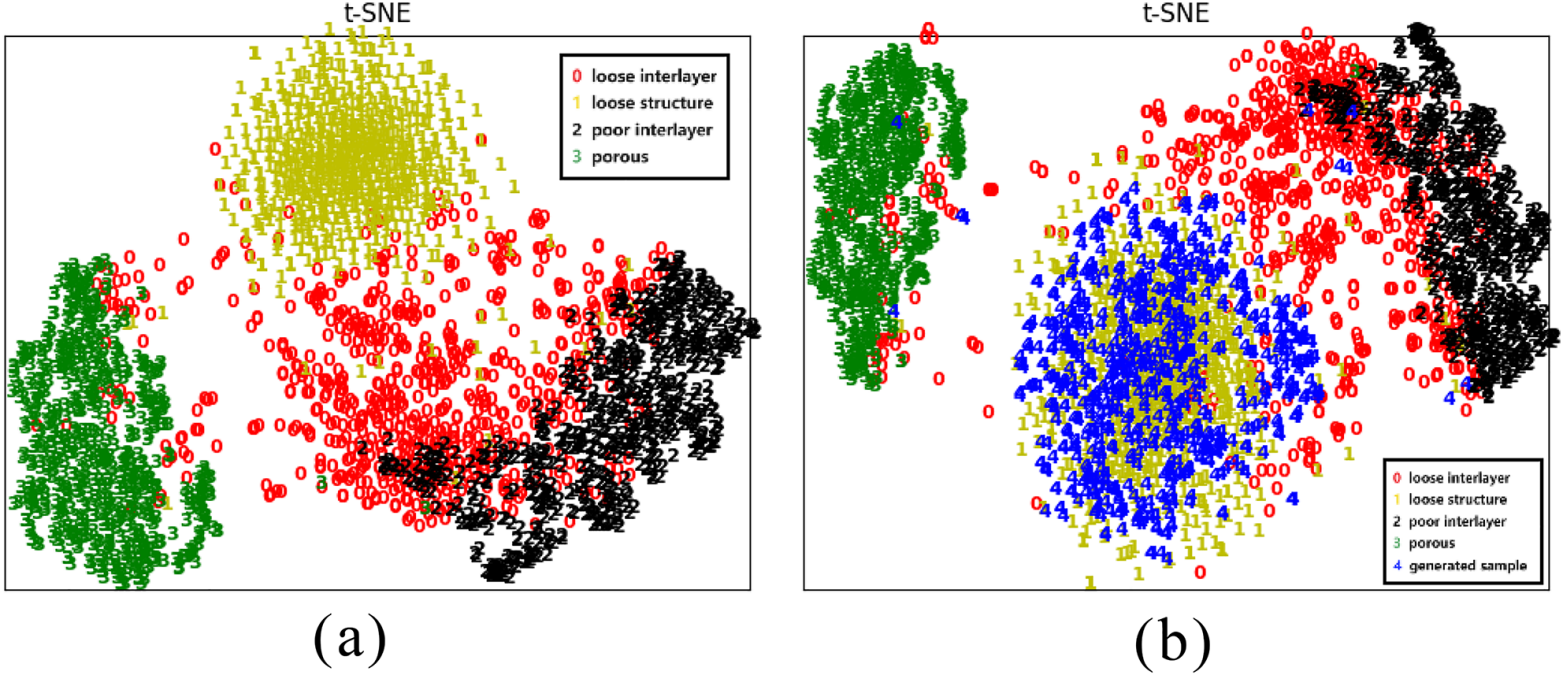
Figure 6. T-SNE 2D plots before and after adding samples (a) original four class samples (b) after adding generated samples (In the Figure, red 0 is loose interlayer, yellow 1 is loose structure, black 2 is poor interlayer, green 3 is porous, and blue 4 is generated sample)

As can be seen from Figure 6(a), the original images can be separated relatively easily in the spatial distribution of the data except for the interlayer looseness, which indicates that there is basically not much problem in the classification of our sample data. Loose interlayer distribution within an independent interval, but since most of the interlayer looseness carries certain characteristics of other damages, or the interlayer looseness is in the early stage of other damages, so it will intersect with the other damages as shown in Figure 6. Figure 6 (b) is the distribution map after adding the structural loosening expansion samples, can be seen from the Figure, to ensure the four types of damages in Figure 6 (a) can be separated, we added samples evenly distributed in the original distribution of damages, basically covering most of the sample distribution space, and there are and its individual generation of samples scattered outside, the proportion of the very low, which effectively illustrates the reliability of the generated data.

**Fig. 7:**
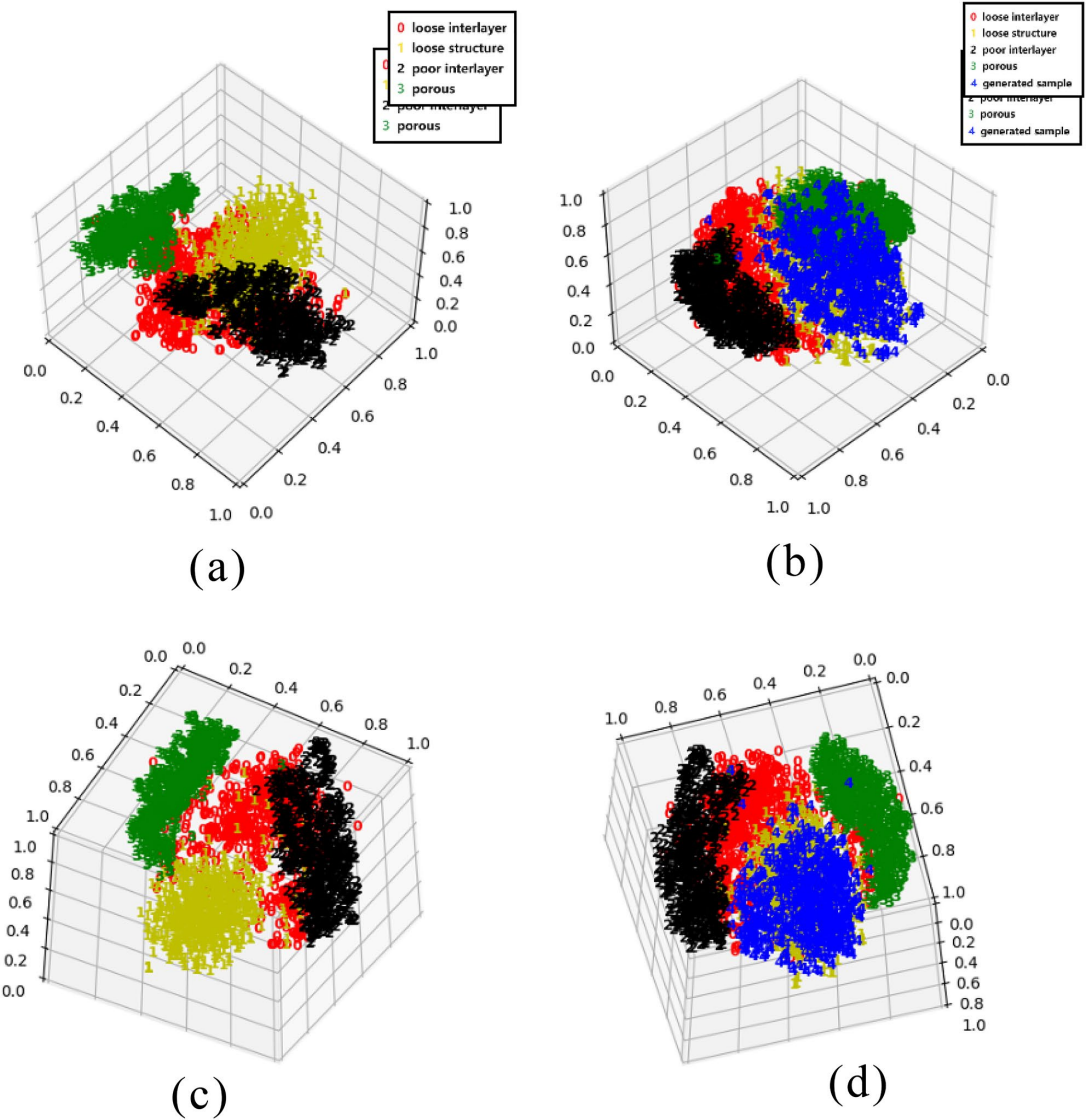
T-SNE 3D plots before and after adding samples (a) Front side of original four-category samples (b) Front side after adding generated samples (c) Side of original four-category samples (b) Side after adding generated samples (red 0 is loose interlayer, yellow 1 is loose structure, black 2 is poor interlayer, green 3 is porous, and blue 4 is generated sample)

From the 3D Figure (Figure 7(a), Figure 7(b)), we can get similar results to those in the 2D Figure, the original four types of samples do not overlap in the distribution of the data space and can be separated more easily. The generated samples tolerate the uniform distribution in the structure of the loose original samples, which can prove that our generated samples are effective. (To see the distribution of data more clearly in space, we rotated the 3D graph and picked out Figure 7 (c) and Figure 7 (d))

Generated samples have an advantage that other centralized traditional methods cannot match - they can fill the original sample space. Getting more information in the original sample space helps the classification network learn the features of the sample better and improve the accuracy.

### Ablation experiments

We divided the experiment into four parts: (1) the original network, (2) the network with modified size and loss function, (3) the network with modified size and loss function and added residuals, an attention mechanism, and (4) the network with modified size and loss function and added residuals, an attention mechanism, and used TTUR. We calculated the FID for the pictures generated by each of these four types of networks with real images to verify the effectiveness of each part. The FID of the pictures generated by each network with the real image is shown in the following Table 2.

**Table 2:** FID results for different networks

Network	Original network	Network with modified size and loss function	Networks with modified size and loss functions and added residuals, attention mechanisms	Modified size and loss functions and added residuals, attention mechanism, used TTUR’s network
FID	88.6044	80.3810	79.2432	74.9581

It can be seen from Table 2 that the FID decreases one by one, because the FID is a measure of the similarity between two multivariate normal distributions using the Fréchet distance, so it can be seen from the table that the images generated by the network after each modification are much closer to the original image in terms of the Fréchet distance, which is a measure of similarity between spaces and is mainly used to measure the similarity between two vector fields of the difference between two vector fields. So we can consider that each modification makes the generated image more similar to the original image to conclude that each modification to the network is effective.

### Classification experiments

The ultimate goal of our dataset scaling and data generation is to improve the classification accuracy to help automated classification and detection afterward, so we use a classification network to classify different scaled datasets and expanded data to verify that our data helps improve the classification accuracy. The classification accuracy is shown in Table 3.

**Table 3:** Classification accuracies for different proportions of datasets

Serial number	1	2	3	4	5	6
Interlayer relaxation	8201	360	360	360	360	360
Interlayer failure	2668	360	360	360	360	360
Porous	360	360	360	360	360	360
Loose structure	98	98	98	98	98	98
Images synthesized using GAN	/	/	82	262	41	/
Images synthesized using other methods	/	/	/	/	41	82
Interlayer relaxation Classification Accuracy	1	0.875	0.975	0.9	0.775	0.95
Interlayer failure Classification Accuracy	0.325	0.8	0.5	0.45	0.7	0.75
Porous Classification Accuracy	0.1	0.75	0.825	0.725	0.75	0.475
Loose structure Classification Accuracy	0.275	0.525	0.75	0.675	0.75	0.475
Overall Accuracy	0.425	0.769	0.762	0.488	0.788	0.744

A brief description of the composition of the dataset: serial number 1 is the complete dataset, which is extremely unbalanced. Serial number 2 is the dataset after deleting some of the excessive data. It becomes more balanced compared to the dataset with serial number 1. Serial number 3 is the dataset with GAN synthesized data added, and the ratio of the number of loosely structured and other classes is 1/2. Serial number 4 is the dataset with GAN synthesized data added, and the ratio of the number of loosely structured and other classes is 1/1. Serial number 5 is the dataset with GAN synthesized data and traditional method synthesized data added, and the ratio of the number of loosely structured and other classes is 1/2. The serial number 6 is the dataset with only traditional method synthesized data added, and the ratio of the number of structurally loose and other classes of data after addition is 1/2.

They are compared one by one below to get the results we want.

**Table 4:** Comparison of classification results for 1 and 2

Serial number	Interlayer relaxation	Interlayer failure	Porous	Loose structure	Interlayer relaxation Classification Accuracy	Interlayer failure Classification Accuracy	Porous Classification Accuracy	Loose structure Classification Accuracy	Overall Accuracy
1	8201	2668	360	98	100%	32.5%	10%	27.5%	42.5%
2	360	360	360	98	87.5%	80%	75%	52.5%	76.9%

As we can see from Table 4, the overall classification accuracy is very low in the case where the original dataset is extremely unbalanced in each class. This is because even if the network classifies all types into “Interlayer relaxation”, the training accuracy is not very low. However, the testing accuracy is very poor. We make the whole data a bit more balanced by removing the data in “Interlayer relaxation” and “Interlayer failure”. You can see that after the above processing, the accuracy of “Interlayer failure”, “Porous” and “Loose structure” are all improved, the accuracy of " Interlayer failure” is improved by 47.5%, the accuracy of " Porous” is improved by 65%, and the accuracy of “Loose structure” is improved by 25%. The overall test accuracy improvement is 34.4%.

Table 4 illustrates that balancing each type of data is effective in improving accuracy.

**Table 5:** Comparison of classification results for 2 and 3

Serial number	Interlayer relaxation	Interlayer failure	Porous	Loose structure	Images synthesized using GAN	Interlayer relaxation Classification Accuracy	Interlayer failure Classification Accuracy	Porous Classification Accuracy	Loose structure Classification Accuracy	Overall Accuracy
2	360	360	360	98	/	87.5%	80%	75%	52.5%	76.9%
3	360	360	360	98	82	97.5%	50%	82.5%	75%	76.2%

Table 5 shows the classification results after adding GAN synthesized data. We can see from the table that after adding a certain number of “Loose structure” data reached 180, and the ratio of other types is 1/2.

Compared with the previous balanced data, the overall accuracy is basically the same. The accuracy of other types has risen and fallen, but the classification accuracy of category “Loose structure” has increased by 22.5%, which shows that adding a certain amount of generated data is effective.

**Table 6:** Comparison of classification results for 3 and 4

Serial number	Interlayer relaxation	Interlayer failure	Porous	Loose structure	Images synthesized using GAN	Interlayer relaxation Classification Accuracy	Interlayer failure Classification Accuracy	Porous Classification Accuracy	Loose structure Classification Accuracy	Overall Accuracy
3	360	360	360	98	82	97.5%	50%	82.5%	75%	76.2%
4	360	360	360	98	262	90%	45%	72.5%	67.5%	68.8%

Table 6 shows the classification results after adding different amounts of GAN synthesized data. We generated more data to add to the dataset so that the class “Loose structure” and the other three classes reach the same amount. Although the data reached the most balanced state, the classification accuracy decreased. Each category has a different degree of decline, and the total precision drops by 7.4%.

We can conclude that over-adding GAN-synthesized data will instead have an adverse effect on the classification accuracy. So the next experiments are all about adding the class a data to the proportions in Table 5.

**Table 7:** Comparison of classification results for 3 and 5

Serial number	Interlayer relaxation	Interlayer failure	Porous	Loose structure	Images synthesized using GAN	Images synthesized using other methods	Interlayer relaxation Classification Accuracy	Interlayer failure Classification Accuracy	Porous Classification Accuracy	Loose structure Classification Accuracy	Overall Accuracy
3	360	360	360	98	82	/	97.5%	50%	82.5%	75%	76.2%
5	360	360	360	98	41	41	77.5%	70%	75%	92.5%	78.8%

Table 7 shows the results obtained with two different kinds of data added. The first one is the data synthesized by all added GAN. The second one is the data synthesized by adding half of the GAN and half of the data synthesized by other methods. In the second case the classification accuracy of loose structure reached the highest in this paper of 92.5%. The classification accuracy of interlayer relaxation decreased by 20%. The classification accuracy of interlayer failure increased by 20%. The classification accuracy of porous decreased by 7.5%. The overall accuracy reached 78.8%. This is an improvement of 2.6% relative to the previous.

With our goal of trying to improve the overall classification accuracy and improve the classification accuracy of loose structure, the second type of method is our most recommended method.

**Table 8:** Comparison of classification results for 3 and 6

Serial number	Interlayer relaxation	Interlayer failure	Porous	Loose structure	Images synthesized using GAN	Images synthesized using other methods	Interlayer relaxation Classification Accuracy	Interlayer failure Classification Accuracy	Porous Classification Accuracy	Loose structure Classification Accuracy	Overall Accuracy
3	360	360	360	98	82	/	97.5%	50%	82.5%	75%	76.2%
6	360	360	360	98	/	82	95%	75%	47.5%	80%	74.4%

Table 8 shows the comparison between the data synthesized by adding GAN only and the data synthesized by adding other methods only. From the table, it can be seen that the other methods are also superior, and the classification accuracy of the loose structure class is improved by 5%. However, the overall accuracy decreased by 1.8%. the classification accuracy of the porous class declined more seriously, by 35%. Only the loose structure class is a little bit more accurate than the GAN method. After comprehensive analysis, we believe that this method is not as effective as the GAN method.

**Table 9:** Comparison of classification results for 5 and 6

Serial number	Interlayer relaxation	Interlayer failure	Porous	Loose structure	Images synthesized using GAN	Images synthesized using other methods	Interlayer relaxation Classification Accuracy	Interlayer failure Classification Accuracy	Porous Classification Accuracy	Loose structure Classification Accuracy	Overall Accuracy
5	360	360	360	98	41	41	0.775	0.7	0.75	0.925	0.788
6	360	360	360	98	/	82	0.95	0.75	0.475	0.8	0.744

As can be seen from Table 9, the results of adding only the data expanded by other methods are far inferior to the results of the methods recommended in this paper. The classification accuracies for all four categories are above 70%. the classification accuracy of Loose structure has increased again by 12.5–92.5% in relative terms. The overall accuracy increased by 4.4–78.8%.

Based on the combined analysis of the results in Tables 4, 5, 6, 7 and 8, and Table 9, the best results were produced by adding both the data synthesized by the GAN and the data synthesized by the other approaches. There is no way to achieve this result by adding either of the approaches alone. Adding GAN alone is slightly better than adding the data synthesized by the other methods alone. Adding the right amount of data is better than adding too much data.

## Conclusion

This paper proposes a GAN-based improvement network to improve the ground-penetrating radar image data imbalance problem; the imbalance problem can be solved to a certain extent by simply deleting redundant data, but the effect is generally unsatisfactory; this paper solves the problem by introducing a new network, the introduced network consists of the encoder, generating the antagonistic network, and the generator and the discriminator are added with spectral normalization, using the least squares loss function, adding a self-attention mechanism, adding an upsampling module, using a dual time scale update rule, etc. The T-SNE algorithm can be used to verify that the data distribution of the generated data is in the original data space, and the FID result proves that the effect of the improved network is better than the initial network. We also use the traditional expansion method, which confirms that no single method is the optimal solution and that using both of them together can achieve better results. In terms of classification accuracy, compared with the original dataset, the classification accuracy of a loosely structured single class can be improved by up to 65%, and the overall classification accuracy can be improved by up to 36.3%.

Our framework has the potential to be extended to other geophysical data problems as well. In our future research, we will improve our approach to accommodate multiple target scenarios. In addition, different target classes will be considered more comprehensively in our follow-up work.

## Data Availability

The dataset generated and analyzed during the current research period belongs to the company’s internal data and has not yet been publicly disclosed but are available from the corresponding author on reasonable request.

## Declarations

The authors are grateful to the key project of National Natural Science Foundation of China(No: 41930112).
